# Editorial: The Adaptive Value of Languages: Non-linguistic Causes of Language Diversity

**DOI:** 10.3389/fpsyg.2018.01827

**Published:** 2018-09-28

**Authors:** Antonio Benítez-Burraco, Steven Moran

**Affiliations:** ^1^Department of Spanish, Linguistics, and Theory of Literature, University of Seville, Seville, Spain; ^2^Department of Comparative Linguistics, University of Zurich, Zürich, Switzerland

**Keywords:** language diversity, cultural evolution, language evolution, adaptive value of languages, language change, ecolinguistics

The goal of this volume is to shed light on the non-linguistic causes of language diversity, and particularly, to explore the possibility that some aspects of the structure of languages result from an adaptation to the natural and/or human-made environment. Variation is pervasive in language. The languages we speak are not homogeneous. They change, both structurally and functionally, from one social group to another, from children to adults, from men to women, from one ethnic group to another, not to mention through historical, and evolutionary time. Moreover, the context in which conversational exchanges take place also affects the structure and the pattern of usage across languages. Besides social variation, geography also accounts for aspects of the variation observed within languages. The differential dispersal of linguistic features across geographically-defined areas usually results in different dialects of one language spoken across the whole distribution area of the language. Ultimately, each person acquires and makes use of a subtly different version of their mother tongue. All of this is very familiar, and over the years, linguists have learnt that these aspects of linguistic variation result from linguistic and extralinguistic factors are constrained in systematic ways, to the extent that they can be described by the right mixture of general principles and statistical biases (e.g. Labov, 2001).

In this Research Topic, we have put the focus on macrovariation across languages from a typological perspective, instead of microvariation within languages, because this aspect of language diversity has been quite satisfactorily characterized by sociolinguists, dialectologists and experts in discourse analysis. When we examine variation at this macro level, we soon realize that thousands of languages are spoken across the world and that they are endowed with distinctive, sometimes idiosyncratic, phonologies, morphologies, and grammars. These aspects of linguistic variation seem to be constrained as well, and we have equally learnt to characterize them in terms of a mixture of common principles and dimensions where languages can differ one from another (e.g., Baker, 2001). Nonetheless, it is not clear what are the causes of this variation. If we put aside the lexicon, which is generally acknowledged to serve as a reservoir for relevant cultural features of the society speaking the language, the twentieth century consensus has been that all languages are roughly equal in terms of overall complexity and that aspects of languages known to vary result from random drift or internally-motivated changes in language structure (Fromkin and Rodman, 1983; Dixon, 1997). To a great extent, this consensus is based on the assumption that human cognition is similarly configured in all human beings, and therefore, that the human faculty for language is uniform within the species (Chomsky, 1965, 1980; Moro, 2008). In the sixties, this assumption crystallized in the Chomskyan hypothesis of the “Universal Grammar.”

This is not exact. In truth, there is also a high degree of variation “at the bottom,” namely, regarding the biological underpinnings of the faculty that enables us to acquire and use languages (let's call it, more neutrally, our *language-readiness*). Accordingly, different language modalities (signed vs. spoken) do exist and can co-exist in the mind of the same user (Emmorey and McCullough, 2009). Additionally, the scores obtained in psycholinguistic tasks change from one person to another across the normal population (Fenson et al., 2000). Language disorders are the extreme of this kind of variation (Benítez-Burraco, 2016). Likewise, language developmental milestones are achieved at different times by children, relying on cognitive abilities that also vary from one to another (Bates et al., 1988; Dehaene et al., 1997). Additionally, the brain areas involved in language processing change, to some extent, from one individual to another (Fedorenko and Kanwisher, 2009; Prat and Just, 2011). Finally, many different genes (not just one or a handful) regulate the development of the brain areas important for language and many of them have functional variants that affect language processing in the neurotypical population (see Benítez-Burraco, 2009 for an overview). Surely, robust biological mechanisms exist as well that channel all this variation, to the extent that a similar faculty of language emerges in all human beings at the end of development, pathologies aside). Although the factors involved are different by nature, this does not differ from the convergence of all speakers of a particular language on a similar interiorized grammar in spite of having being reared in linguistic environments that are not identical.

Likewise, it seems now that languages also differ regarding their global complexity. The complexity of languages can increase, for instance, as a result of specific linguistic processes, like grammaticalization, which increases the number of categories or the number of irregularities (Givón, 1979). More importantly, the overall language complexity, as well as the complexity of specific components of the languages' grammars, can perhaps be explained by extralinguistic factors as well. Accordingly, language complexity has been found to correlate with features of the social environment impacting on language contact and language acquisition. For example, it seems to be greater when the language has more native speakers, when speakers are not involved in frequent cross-cultural exchanges, and when they are isolated (Bolender, 2007; McWhorter, 2007; Wray and Grace, 2007; Lupyan and Dale, 2010; Trudgill, 2011). As for another example, it has been claimed that a positive correlation exists between population size and phoneme inventory size (Hay and Bauer, 2007, but see Moran et al., 2012 for an opposite view). Eventually, core properties of human languages, like duality of patterning, have been argued to emerge as a result of iterative learning and cultural evolution, as nicely illustrated by research in village sign languages (Sandler et al., 2005) or in language evolution (Fleming, 2017). In a similar vein, language structure is also thought to be influenced on a long timescale by the physical environment, either directly or indirectly, via its effect on social structures. Familiar examples are the negative effect of dry climates on tone usage and the number of vowels (Everett et al., 2015), or of dense vegetation on sounds characterized by lower frequencies (Maddieson, 2011; Maddieson and Coupé, 2015). More generally, global language diversity has been claimed to negatively correlate with the ecological risk, that is, the amount of variation which people face in their food supply over time (Nettle, 1998). Similarly, the number of phyla or stocks has been suggested to negatively correlates with the time of occupancy of a territory (Nettle, 1998). Overall, it seems desirable to have a better knowledge of current patterns of linguistic diversity across the world, and particularly, of the ecological and socio-cultural factors that correlate with (and ideally, explain) aspects of this diversity. From an evolutionary perspective, we wish to know more about the adaptive value of language diversity and how it emerges over time as the physical, social, and cultural environment becomes modified. Several of the papers of this Research Topic explore this kind of correlation (and causation). Ultimately, we expect that these and other similar studies cast light as well onto some aspects of the deep evolution of language (and languages), provided that niche construction (perhaps via human self-domestication) has proven to account for aspects of language complexity via cultural evolution (Benítez-Burraco et al., 2016) and because some aspects of languages seem to be an adaptation to ecological, social, or cultural niches.

Finally, language complexity is also expected to be influenced by cognitive patterns, for instance, if some kind of processing preference biases language learning and use, and ultimately, what becomes grammaticalized (Bornkessel-Schlesewsky and Schlesewsky, 2009). [Note the other way around is also true, because aspects of language that are more costly to process and learn might favor the creation of “cognitive gadgets” through modifications in learning and data-acquisition mechanisms (Clarke and Heyes, 2017)]. More generally, recent research has concluded that cognitive differences among human populations do exist and are in part due to genetic changes in response to environmental factors, and not only to cultural or sociological forces (Winegard et al., 2017). Similarly, our “language genotype” (that is, the set of genes involved in the development and functioning of brain areas recruited for language processing) is not homogeneous either, with variants of specific genes contributing to normal variation in speech and language abilities (Deriziotis and Fisher, 2017). Accordingly, we could speculate about certain gene alleles influencing on aspects of languages that are known to vary, like phonology or morphosyntax. Again, this effect might be direct, if the involved genes contribute, for instance, to aspects of our vocal behavior. But most plausibly, we should expect that the effect is indirect, if specific alleles bias language acquisition or processing in some subtle ways, ultimately impacting on language change through iterated cultural transmission (Dediu, 2008, 2011). It is clear then that it seems desirable to better understand the complex interaction between genes, cognition, and the environment, and its effects on language diversity, both in the present-day populations and in the remote prehistory. In this sense, gene-culture co-evolution is expected to account for crucial aspects of language diversity too.

In this volume we bring together 12 contributions from 25 leading scholars in different research areas of interest for the questions we have highlighted above. Three of the papers discuss important theoretical and methodological issues. Mendívil-Giró adds a note of caution regarding the sources of language variability. According to his view, it is the structure of the brain/mind that mostly affects language structure and we should make dependent of this circumstance any putative effect of the environment on how languages are built. Roberts presents a *maximum robustness* approach for studying adaptation in language. The method is a causal, incremental and robust approach aimed at testing hypotheses and identifying linguistic adaptation patterns in a world of increasing data, methods, and computational power. He addresses how to formalize a theory and how to identify criteria for integrating results from different approaches and methods into clear hypothesis testing and results assessment. Finally, the paper by Coupé focuses on optimal statistical tools for analyzing potential correlations between linguistic and extralinguistic variables. In particular, he discusses several techniques that help modeling data that are not analyzable with simpler linear regression models, including linear mixed-effects regression models (LMM), generalized linear mixed-effects models (GLMM), generalized additive models (GAM), and generalized additive models for location, scale, and shape (GAMLSS), which allow one to circumvent the limitations of commons distributions.

Turning to the papers exploring correlations between linguistic and extralinguistic variables, two of them address potential links between aspects of the physical environment and features of languages. Maddieson has found that the proportion of sonority vs. obstruency is higher in languages spoken in warmer climes. Interestingly, he suggests that given the highly malleable nature of the phonological structure of human languages, the time scale in which environmental factors influence the phonological make up of languages is acting at a scale faster than previously put forward in the literature. Likewise, Everett shows evidence for a positive association between reduced ambient humidity and reduced vowel-usage rates in a large sample of the world's languages. Importantly, some physiological evidence, involving larynx behavior, is presented to account for the observed correlation. Overall, the effect of the environment on languages' phonologies is controversial and we should be cautious with such approaches and scrupulous of the results, as stressed by Roberts and Maddieson.

Four other papers focus on the links between language diversity and sociological features. Nichols examines the effect of language mixing on the emergence of what she calls “linguistic attractors,” that is, linguistic items, and features that are preferred by languages in their evolution. As she highlights, the emergence of linguistic attractors is linked to specific demographic, sociological, cultural, and environmental factors. Greenhill et al. contribute to the long and ongoing debate of whether population size has an observable effect on language change. In particular, they ask whether rates of lexical replacement in three large language families (Austronesian, Indo-European and the Bantu subfamily of Niger-Congo) are affected by speaker population size. Their results show an effect that does not generalize across families. Greenhill et al.'s paper is also important as well because it highlights the differences between historical transmission of languages and the evolution of biological organisms. Whereas evolutionary theory makes clear predictions of rates and patterns of genetic change in regard to population size, it seems that language change may be driven by different mechanisms. Sinnemäki and Di Garbo focus on a related effect of the sociolinguistic environment on language structure, namely, the effect of the number of native speakers and the proportion of adult second language learners, which have been claimed to have an impact on language complexity, and particularly, on morphological complexity (Lupyan and Dale, 2010). Their data suggest that different sociolinguistic variables might affect different grammatical features differently. Importantly, they argue that modeling together several sociolinguistic features favors detecting possible adaptation of linguistic structure to the sociolinguistic environment. Lastly, Schembri et al. explore the links between the social environment and language structure sign languages. This is important provided that sign languages are endowed with the same structural features and properties as oral languages. What Schembri et al. have found is that sign languages change might support the view that morphological complexity depends on social features of the speech community. Nonetheless, they warn against a direct effect of population size and network density on language complexity, which seems to depend as well on how and when the language is acquired and its degree of contact with other language modalities.

Finally, three papers deal with the cognitive aspects of language variation. González-Perilli et al. study color object perception in two different Spanish-speaking populations, and show that Uruguayans, who use single words for two shades of blue, are more accurate at distinguishing between light blue and dark blue in a color stimuli perception task than are Spaniards, who use compound terms. These findings add to the ongoing debate of whether language and culture affect how individuals organize and process information from their world experience. Linguistic relativity effects are disputed by researchers, but there is much evidence for them across different cognitive domains and languages, including spatial cognition, and color recognition. Kempe and Brooks raise two important points of caution regarding the finding by Lupyan and Dale (2010) that morphological complexity is negatively correlated with population size. First is the need to improve our characterization (and understanding) of language complexity, if we want to properly address the questions of whether languages are equally complex and whether languages remain so by compensating for complexity in different subsystems of grammar [see (Moran and Blasi, 2014), and inter alia, for an overview]. Regarding morphological complexity, which is the focus of Kempe and Brooks' paper, the authors suggest that operationalizing morphological complexity based on combined informational value of morphological cues in the languages might be the best choice to capture the links between language processing and language change. Second, Kempe and Brooks also warn against the view that the cognitive limitations of children support mechanisms beneficial for learning of complex morphology relative to adults. The authors argue convincingly that the difference in learning strategies by child and adult learners needs to have a more solid empirical foundation in which it is crucial to define morphological complexity with operationalizations that are cognitively-based. Lastly, the paper by Toya and Hashimoto aims to identify the environmental triggers and the evolutionary path of recursive combination, thought to be a human-specific ability and a core operation in human languages. They rely on a learning game approach. Their results suggest that recursive combination is adaptive because it results in more robust production mechanisms and more diversified products, a lesson that can be extended to material culture, human cognition, and language.

This volume contributes to the exciting challenges of disentangling the effect of the environment on language structure and complexity, and ultimately, helps us to form a better understanding of the nature and evolution of human language.

## Author contributions

AB-B and SM conceived and proposed this Research Topic and contributed equally to the editing duties and work.

### Conflict of interest statement

The authors declare that the research was conducted in the absence of any commercial or financial relationships that could be construed as a potential conflict of interest.
